# Variability of respiratory rate measurements in children suspected with non‐severe pneumonia in north‐east Tanzania

**DOI:** 10.1111/tmi.12814

**Published:** 2016-12-12

**Authors:** Florida Muro, Neema Mosha, Helena Hildenwall, Frank Mtei, Nicole Harrison, David Schellenberg, Raimos Olomi, Hugh Reyburn, Jim Todd

**Affiliations:** ^1^ Kilimanjaro Christian Medical University CollegeMoshiTanzania; ^2^ Kilimanjaro Christian Medical CentreMoshiTanzania; ^3^ Global Health – Health System and Policy Research GroupKarolinska InstitutetStockholmSweden; ^4^ Joint Malaria ProgrammeKilimanjaro Christian Medical CentreMoshiTanzania; ^5^ Department of Medicine IMedical University of ViennaViennaAustria; ^6^ London School of Hygiene & Tropical MedicineLondonUK

**Keywords:** pneumonia, respiratory rate, children, video, variability, integrated management of childhood illness

## Abstract

**Objective:**

Measurement of respiratory rate is an important clinical sign in the diagnosis of pneumonia but suffers from interobserver variation. Here, we assess the use of video recordings as a quality assurance tool that could be useful both in research and in training of staff.

**Methods:**

Respiratory rates (RR) were recorded in children aged 2–59 months presenting with cough or difficulty breathing at two busy outpatient clinics in Tanzania. Measurements were repeated at 10‐min intervals in a quiet environment with simultaneous video recordings that were independently reviewed by two paediatricians.

**Results:**

Eight hundred and fifty‐nine videos were sent to two paediatricians; 148 (17.2%) were considered unreadable by one or both. For the 711 (82.8%) videos that were readable by both paediatricians, there was perfect agreement for the presence of raised RR with a kappa value (*κ*) of 0.85 (*P* < 0.001); and in 476 (66.9%) cases, both paediatricians agreed on the RR within 2 breaths per minute (±2 bpm). A reported illness of 5 days or more was associated with unreadable video recordings (OR = 3.44, CI: 1.5–6.08; *P* < 0.001). The multilevel model showed that differences between observers accounted for only 13% of the variability in RR.

**Conclusion:**

Video recordings are reliable tools for quality assurance of RR measurements in children with suspected pneumonia. Videos with a clear view of respiratory movements may also be useful in training primary healthcare staff.

## Abstract (fr)

**Objectif:**

La mesure de la fréquence respiratoire est un paramètre clinique important dans le diagnostic de la pneumonie mais souffre de variations inter‐observateurs. Nous évaluons ici l'utilisation des enregistrements vidéo comme un outil d'assurance de la qualité qui pourrait être utile tant pour la recherche que pour la formation du personnel.

**Méthodes:**

Les fréquences respiratoires (FR) ont été enregistrées chez les enfants âgés de 2 à 59 mois présentant de la toux ou des difficultés respiratoires dans deux cliniques ambulatoires chargées, en Tanzanie. Les mesures ont été répétées à des intervalles de 10 minutes dans un environnement silencieux avec des enregistrements vidéo simultanés qui ont été examinés indépendamment par deux pédiatres.

**Résultats:**

859 vidéos ont été envoyées à 2 pédiatres. 148 (17.2%) vidéos ont été jugées illisibles par l'un ou les deux. Pour les 711 vidéos (82.8%) lisibles par les deux pédiatres, il y avait un accord parfait pour la présence de FR élevée avec une valeur kappa (*κ*) de 0.85 (*P* < 0.001); et dans 476 cas (66.9%), les deux pédiatres ont été d'accord sur la FR à 2 respirations par minute près (±2 bpm). Une maladie rapportée de 5 jours ou plus était associée à des enregistrements vidéo illisibles (OR = 3.44; IC: 1.5–6.08; *P* < 0.001). Le modèle à multi niveaux a montré que les différences entre les observateurs ne représentaient que 13% de la variabilité de la FR.

**Conclusion:**

Les enregistrements vidéo sont des outils fiables pour l'assurance de la qualité des mesures de la FR chez les enfants souffrant de pneumonie. Des vidéos avec une vue claire des mouvements respiratoires peuvent également être utiles dans la formation du personnel des soins de santé primaires.

**Mots‐clés:**

pneumonie; fréquences respiratoires; enfants; Video; Variabilité.

## Abstract (es)

**Objetivo:**

La frecuencia respiratoria es un signo clínico importante en el diagnóstico de la neumonía pero se ve afectada por la variación interobservador. Hemos evaluado el uso de grabaciones de vídeo como una herramienta de control de calidad que podría ser útil tanto en investigación como para entrenar al personal sanitario.

**Métodos:**

Se midió la frecuencia respiratoria (FR) en niños con edades entre los 2–59 meses que se presentaban con tos o dificultad respiratoria en las consultas externas de dos clínicas de Tanzania. Las medidas se repetían en intervalos de 10 minutos, en un ambiente silencioso, con grabaciones simultáneas de video que eran revisadas de forma independiente por dos pediatras.

**Resultados:**

Se enviaron 859 videos a 2 pediatras. 148 (17.2%) se consideraron ilegibles por uno o por ambos. En los 711 (82.8%) considerados legibles por los dos pediatras, había una concordancia perfecta de FR aumentada con un valor kappa (*κ)* de 0.85 (*P* < 0.001); y en 476 (66.9%) de los casos ambos pediatras estaban de acuerdo en la FR con la frecuencia dentro de un rango de 2 respiraciones por minuto (±2 rpm). Una enfermedad reportada de 5 días o más estaba asociada con una grabación de video ilegible (OR = 3.44, CI 1.5–6.08; *P* < 0.001). El modelo multinivel demostraba que las diferencias entre observadores eran responsables de solamente un 13% de la variabilidad en FR.

**Conclusión:**

Las grabaciones en video son herramientas fiables para asegurar la calidad de las medidas de FR en niños con sospecha de neumonía. Los videos con una imagen clara de los movimientos respiratorios también podrían ser útiles para entrenar a personal sanitario de atención primaria.

**Palabras clave:**

frecuencia respiratoria**;** neumonía; Variabilidad; niños; video.

## Introduction

Childhood pneumonia constitutes the largest single cause of child mortality globally 1, a burden that could be substantially reduced by timely diagnosis and antibiotic treatment early in the illness. Consequently, the WHO case definition of pneumonia aims at high sensitivity whereby children with a cough or difficulty in breathing and a raised respiratory rate (for age cut‐offs) are classified as having ‘non‐severe’ or, more recently, ‘fast‐breathing’ pneumonia, and should be treated with oral antibiotics 2, 3. However, the measurement of respiratory rate in young children is often problematic. Other clinical states such as fever or agitation may confound the association of fast breathing with pneumonia 4. There is evidence of interobserver differences in counting respiratory rates that may account for substantial misclassification of children with suspected pneumonia 5, 6. A number of timing devices have been proposed to improve accuracy of respiratory rate measurement such as audible signals, variations in timing period and tapping a smart phone screen at each respiration 7, 8, 9, 10. These tools have been assessed using standard video recordings of respiratory movements, but the validation appears to be based on selection of videos where respiratory rate was clearly seen by an expert. The problems of ‘real‐life’ measurements have been highlighted by English *et al*. in a review of video recordings of common paediatric clinical signs; both experts and untrained staff agreed where the sign was clearly visible and abnormal, but agreement was no better than chance where the sign was borderline or difficult to see 11. This study did not include measurements of respiratory rate and it is not clear how common such difficulties are with respect to the measurement of respiratory rate during the process of routine care.

In spite of recommendations that measurement of the respiratory rate (RR) should be performed in a calm child, there is evidence of substantial errors in its recording that result in both overtreatment and missed diagnoses. These errors may be reduced by improved training of clinical staff in counting RR, but such training is constrained by the absence of an accessible ‘gold standard’ of measurement.

In a study of the influence of the clinic environment on paediatric respiratory rate, video recordings of children's respiratory movements were independently reviewed by two experienced paediatricians as a quality control exercise. We analysed these results to identify criteria that would be expected of a valid gold standard, that is that video recordings were judged to be readable and respiratory counts reached good levels of agreement.

## Methods

### Study area and participants

The study was conducted in the outpatient departments of two district hospitals in north‐eastern Tanzania, one serving a predominantly rural population and the second serving an urban population. Details of the study participants recruitment and enrolment have been published elsewhere 12. In summary, children aged 2–59 months presenting at outpatient clinics with cough or difficulty breathing were screened for malaria using Paracheck™ rapid test for malaria and haemoglobin levels using Hemocue™. They were also assessed for use of anticonvulsant or sedative medication in the previous 24 h, signs of severe illness including lower chest wall in‐drawing and evidence of chronic illness including asthma, and malnutrition.

The Tanzanian national guideline abides by the WHO Integrated Management of Childhood illness (IMCI) recommendations for the management of common childhood illnesses such as pneumonia 2.

Caregivers of children gave written informed consent to participate in the study. We recorded the medical history and basic socio‐demographic characteristics of all enrolled children, followed by a careful physical examination including measurement of respiratory rate (RR) over one minute by research physician.

After the initial assessment at the outpatient clinic, children were transferred to a quiet room where the research nurse observed them for an hour. Every 10 min, an RR count was taken using a timer with an accompanying video recording of the child's chest movement. Each video displayed the date, time and study identification number (IDN) of the subject. Two Panasonic Lumix FS45 cameras equipped with 16 megapixels, 5× optical zoom and high definition (HD) video were used at each study site to film the child's chest (exposed). Using tripods, the camera was positioned aiming downwards on the child's chest and moved to a required angle when necessary.

The camera was switched on once the child was settled, calm or stable. If not, the child's state was documented (moving, sleeping, feeding and agitated) on the case report form. Agitation was defined as any movement and/or crying that interrupted the counting or recording of the RR. Any RR count or video recording that failed was documented and then related to the participant's characteristics. In circumstances where children could not settle at all, or the equipment malfunctioned, video recording was stopped and no video was available for later review for that child at that time point. The nurse was responsible for both RR count and camera operation and she had been trained on the WHO respiratory rate counting. Respiratory rate counting was started once the clinical state of the child was judged to be stable.

The video recording of child's chest movements was read independently by two consultant paediatricians experienced in managing childhood pneumonia and teaching staff on the use of IMCI guidelines. If both paediatricians could read the video, it was classified as readable, and those not readable by either of the paediatricians were classified as unreadable. Video ‘readability’ was defined *a priori* as the paediatrician being able to view the child chest movements and count RR for 1 whole minute either easily or with some difficulty. A video counted as not readable if the paediatrician could not view and count RR for 1 whole minute.

### Data management and analysis

Data were double‐entered into a Microsoft Access database and statistical analyses performed using stata version 12.0 software. Non‐severe pneumonia was defined as cough or difficulty breathing with a raised RR for age, that is ≥50 breaths per minute (bpm) for those aged 2–12 months and ≥40 bpm for those aged 12–59 months.

The mean RR and 95% confidence intervals (95% CI) in RR were calculated at each time point, using the data from all children measured by the three observers – research nurse, paediatrician 1 and paediatrician 2.

Across all time points when both paediatricians were able to read the video recording of the child's chest movement, three measures of agreement were calculated. A kappa statistic (*κ*) was calculated for the agreement on the presence of raised RR between the two paediatricians. The agreement scores ranges from 1 for a perfect agreement to 0 for no agreement beyond chance using the classification from Landis and Koch: 0.0–0.20 signified slight agreement, 0.21–0.40 fair agreement, 0.41–0.60 moderate agreement, 0.61–0.80 substantial agreement and 0.81–1.00 almost perfect agreement 13.

The correlation coefficient was calculated on the RR measures to show the agreement between the two paediatricians in counting the RR. The third measure defined agreement as any RR where the two paediatricians were within 2 breaths per minute (±2 bpm) of each other, which was then analysed as a binary variable using logistic regression to obtain odds ratios (OR) and 95% confidence intervals (95% CI). The ±2 bpm was chosen as representing error that could arise from including or excluding the first or last breath during the 1‐min period of counting.

To assess for variability in RR count, a linear regression model was used to analyse the crude and adjusted effects across three levels of variability: children, time and observers. The coefficients (differences in RR) were calculated for the characteristics of the children, taking into account the variability between children, between time points within children and the differences between the measurements by three observers (nurse and two paediatricians) within time points.

The adjusted effect of the final model includes all the significant independent characteristics at each level. From the final model, we assess the proportion of respiratory rate variability (intraclass correlation coefficient (ICC)) at each level of the model.

### Ethical approval

The study was approved by the Kilimanjaro Christian Medical Centre (KCMC) Research Ethics Committee, Tanzania. We obtained written informed consent from the parent/legal guardian of each child in the study.

## Results

### Characteristics of study participants

Overall 167 children were enrolled of whom 162 (97.0%) had at least one repeat measurement of respiratory rate in quiet settings, and were used in this analysis. The mean age was 7.1 (SD ± 2.8) months in infants and 27.6 (SD ± 12.8) months in children aged 12–59 months. More males (94, 58.0%) than females (68, 42.0%) were enrolled. Other baseline characteristics of these children have been described elsewhere 12.

### Comparison of respiratory rate readings between the research nurse and the two paediatricians

**Figure 1:**
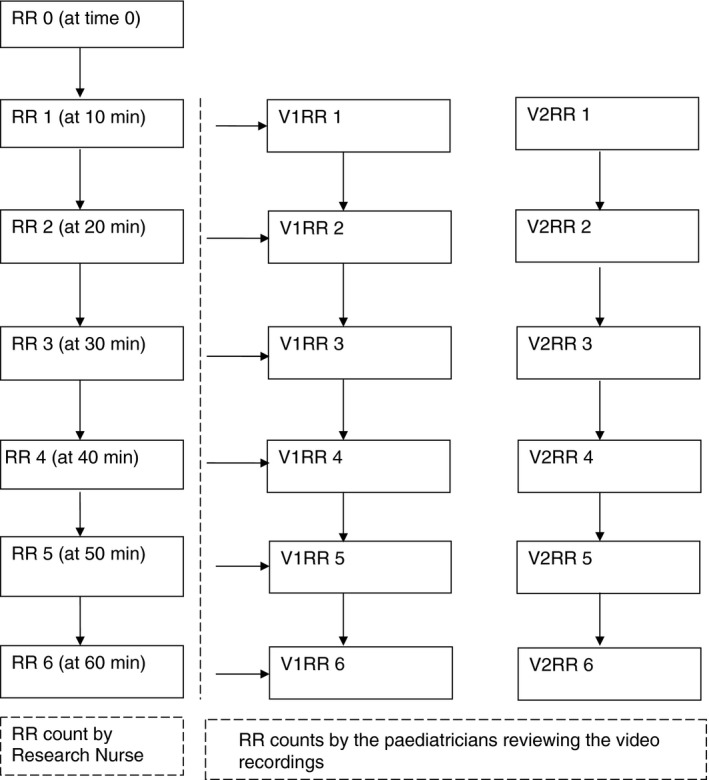
Schematic diagram showing different time points that a child in the study underwent respiratory rate (RR) count over an hour.

*Note*: V1‐video review of RR by paediatrician 1. V2‐video review of RR by paediatrician 2. Video recordings were done at the same time as the RR by the research nurse and the reviews of the video recording of the chest movement were done after data collection by the two paediatricians.

After the initial RR count at the clinic, each study participant had RR counts at six time points in the quiet room. For each time point, there are three RR readings available, one direct measurement by the research nurse and two by two paediatricians from the video recording (Figure 1); 113 video records were incomplete and not available for analysis.

### Readability of the videos by the two paediatricians

There were 859 available videos from 162 children, which were reviewed by the two observer paediatricians. Eighty (8.2%) videos were not readable to paediatrician 1; 142 (14.6%) were not readable to paediatrician 2 (Table 1). In the combined results, 711 videos (82.8%) were readable by both paediatricians and 148 (17.2%) were not (Table 1).

**Table 1:** Description of the children's videos according to the two observer paediatricians

	Easily readable, *n* (%)	Readable with difficult, *n* (%)	Not readable, *n* (%)	No video available, *n* (%)	Total, *N* (%)
Observer 1	597 (61.5)	182 (18.7)	80 (8.2)	113 (11.6)	972 (100)
Observer 2	554 (57.0)	163 (16.8)	142 (14.6)	113 (11.6)	972 (100)
Overall	Readable video recording = 711 (82.8%)		Unreadable video recordings = 148 (17.2%)		859 (100)

Unreadable video recordings include those videos that were available but not readable to either of the observer paediatrician**.**

**Figure 2:**
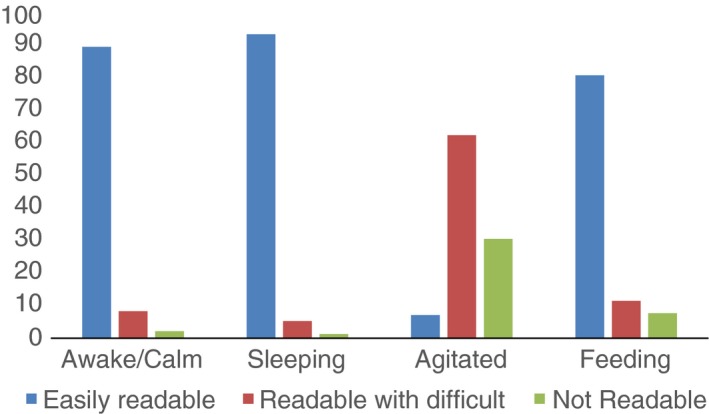
Video quality according to the different child states by video observer (paediatrician 1) *N* = 859.

**Figure 3:**
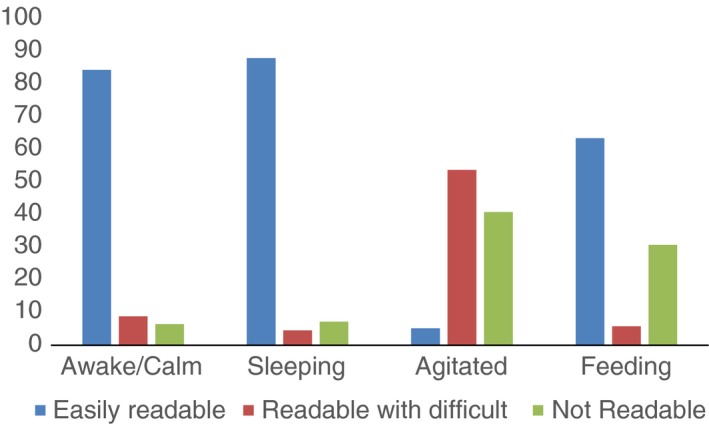
Video quality according to the different child states by video observer (paediatrician 2) *N* = 859.

Looking at the child state, 446 (51.9%) of 859 videos were of children who were awake and calm and 210 (24.4%) videos were of children who were agitated (mild or severe), making video review difficult or impossible (Figures 2 and 3). Of the remaining videos, 152 (17.6%) were of children sleeping and 52 (6.0%) of children feeding.

Of 859 video recordings, 497 (57.9%) were of boys; 196 (29.5%) had fever at baseline examination. There were more readable videos of males (425, 85.5%) than females (286, 79.0%), and of older children (371, 85.7%) than younger children (340, 79.8%) (Table 2). A larger proportion of videos of children who had no fever was readable by both paediatricians (563, 84.9%).

**Table 2:** Proportion of video recordings of children with cough or difficult breathing attending outpatient clinics in Tanzania that were reviewed as readable or not readable by the two paediatricians, *N* = 859

Variable	With readable video (either easily readable or readable with difficulty) by both paediatricians, *n* = 711	With video unable to be read by both paediatricians, *n* = 148	Total *N* = 859 (100)	*P*‐value
Sex				
Female	286 (79.0)	76 (20.9)	362 (100)	**0.010**
Male	425 (85.5)	72 (14.5)	497 (100)	
Age group				
Younger children (<12 months)	340 (79.8)	86 (20.2)	426 (100)	**0.020**
Older children (12–59 months)	371 (85.7)	62 (14.3)	433 (100)	
Temperature at baseline				
Normal (<37.5 °C)	563 (84.9)	100 (15.1)	663 (100)	**0.002**
High (≥37.5 °C)	148 (75.5)	48 (24.5)	196 (100)	
History of rapid breathing				
No	425 (82.1)	93 (17.9)	518 (100)	0.500
Yes	286 (83.9)	55 (16.1)	341 (100)	
Duration of illness				
<5 days	565 (80.7)	135 (19.3)	700 (100)	**0.008**
≥5 days	146 (91.8)	13 (8.2)	159 (100)	
Child's state				
Awake/calm	415 (93.1)	31 (6.9)	446 (100)	**<0.001**
Sleeping	140 (92.7)	11 (7.3)	151 (100)	
Agitated	120 (54.1)	90 (42.9)	210 (100)	
Feeding	36 (69.2)	16 (30.8)	52 (100)	

Unreadable video recordings include those videos that were available but not readable to either of the observer paediatrician**.** Bold *P*‐values indicates significant associations

### Agreement on the RR counts and criteria for non‐severe pneumonia

#### Kappa statistic for the presence of raised respiratory rate for age

In all readable videos, agreement on the presence of a raised RR (non‐severe pneumonia) between the two paediatricians was 95.9% (better than the expected agreement) with kappa value of 0.85 indicating a perfect agreement in assessing non‐severe pneumonia.

#### Correlation coefficient

**Figure 4:**
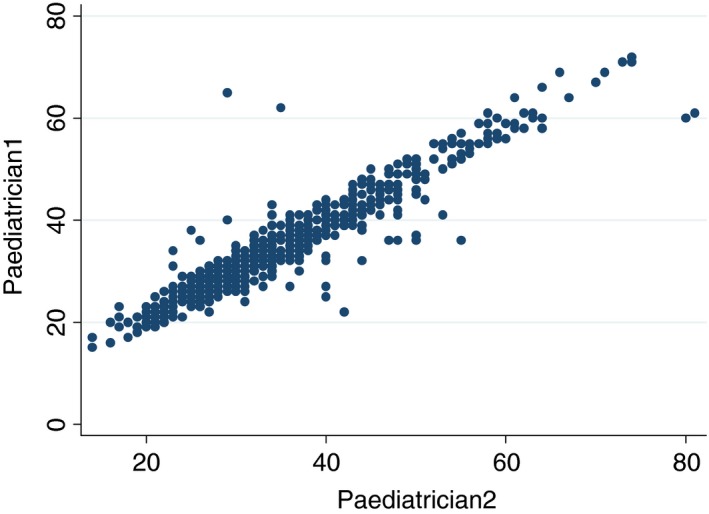
Scatter plot of paediatrician 1 individual RR counts *vs*. paediatrician 2 RR counts for 859 children.

Overall, there was good agreement on the RR count by the two paediatricians (correlation coefficient: 0.94). However, the correlation coefficient may be influenced by RR readings at the higher and lower end of the scale, and Figure 4 shows the graphic representation of variability in RR counts.

#### Agreement within 2 bpm (±2 bpm) for the RR measurement

Allowing a variation of 2 breaths per minute (bpm) as agreement in RR count between the two paediatricians for readable video recordings, there was a 33% disagreement. The expected agreement by chance is 50.2%, so 67% agreement gives a kappa of 0.34, which shows a fair agreement between the two paediatricians.

Table 3 shows a proportion of the 711 video recordings that were readable within ±2 bpm and the univariate factors for agreement between the two paediatricians. In univariate analysis, agreement between the two paediatricians was lower if children were agitated (OR = 0.71, CI: 0.44–1.15; *P* = 0.161) and higher if they were feeding (OR = 1.88, CI: 0.77–4.55, *P* = 0.164). However, they were more likely to agree on RR of *±*2 bpm in children with longer duration of the illness (OR = 3.44, CI: 1.95–6.08; *P* < 0.001).

**Table 3:** Proportion of video recordings of children with cough or difficult breathing attending outpatient clinics in Tanzania that were readable by both observer paediatricians, *N* = 711

Variable	Agreement of ±2 bpm RR by both paediatricians, *N* = 711			*P*‐value
	Yes 476 (66.9%)	No 235 (33.1%)	OR (95% CI)	
Sex				
Male	272 (64.0)	153 (36.0)	1	
Female	204 (71.3)	82 (28.7)	1.23 (0.79–1.90)	0.360
Temperature at baseline				
Normal (<37.5 °C)	375 (66.6)	188 (33.4)	1	
High (≥37.5 °C)	101 (68.2)	47 (31.8)	1.02 (0.61–1.71)	0.944
History of rapid breath				
No	289 (68.0)	136 (32.0)	1	
Yes	187 (65.4)	99 (34.6)	0.98 (0.63–1.52)	0.935
Duration of illness				
<5 days	353 (62.5)	212 (37.5)	1	
≥5 days	123 (84.3)	23 (15.7)	**3.44 (1.95–6.08)**	**<0.001**
Child state				
Awake/calm	280 (67.5)	135 (32.5)	1	
Sleeping	98 (70.0)	42 (30.0)	1.34 (0.82–2.20)	0.248
Agitation	71 (59.2)	49 (40.8)	0.71 (0.44–1.15)	0.161
Feeding	27 (75.0)	9 (25.0)	1.88 (0.77–4.55)	0.164

OR indicates an odds ratio; it is adjusted for age group. CI indicates confidence intervals; bolded OR and CIs indicate significant level at *P*‐value of <0.05. °C indicates degree Celsius.

### Regression model using repeated RR measures by different observers

Random‐effects linear regression was used to characterise how the observed respiratory rate varied with clinical characteristic of the children (Level 1 variables: age, body temperature, duration of illness); the effect of time (Level 2 variables: time since first measurement (in minutes), child's state) and observer (Level 3 variable). The baseline level is the RR at 10 min for children who are awake and calm, when the research nurse recorded the respiratory rate.

Univariate analysis (Table 4) showed a strong evidence of differences in RR by body temperature at baseline (Level 1), the child's state during RR measurement (Level 2) and observers (Level 3). The main difference between the observers was that the two paediatricians who reviewed the video of the child had higher estimate of the RR (coefficient: 0.53 and 0.78) than the nurse who directly measured the RR.

**Table 4:** Univariate and multivariate regression analysis of RR measurement over time by different observers, *N* = 859

Variable	Univariate			Multivariate		
	Coefficient, 95% CI	SE	*P*‐Value	Coefficient, 95% CI	SE	*P*‐Value
Level 1						
Female	0.39 (−2.28 to 3.08)	1.37	0.771			
Age (months)	−0.26 (−0.39 to −0.12)	0.07	**<0.001**	−0.31 (−0.39 to −0.14)	0.04	**<0.001**
Temperature at baseline (°C)	5.77 (2.75 to 8.79)	1.54	**<0.001**	5.82 (3.07 to 8.72)	1.43	**<0.001**
History of rapid breathing	2.70 (−0.01 to 5.37)	1.37	**0.048**	2.83 (0.31 to 5.17)	1.23	**0.022**
Duration of illness	−1.18 (−3.51 to 3.15)	1.69	0.915			
Level 2						
Effect of time (minutes)						
10	Baseline			Baseline		
20	−0.56 (−1.71 to 0.58)	0.58	0.338	−0.04 (−1.15– 1.07)	0.57	0.936
30	−0.46 (−1.60 to 0.68)	0.58	0.432	0.19 (−0.92 to 1.31)	0.57	0.942
40	−0.77 (−1.93 to 0.39)	0.59	0.196	−0.41 (−1.53 to 0.71)	0.57	0.475
50	−0.90 (−2.09 to 0.28)	0.60	0.134	−0.01 (−1.17 to 1.16)	0.59	0.991
60	−1.25 (−2.45 to −0.05)	0.61	**0.041**	−0.62 (−1.79 to 0.55)	0.59	0.300
Child's state						
Awake/calm	Baseline			Baseline		
Sleeping	−3.83 (−4.95 to −2.69)	0.59	**<0.001**	−3.94 (−5.09 to −2.79)	0.59	**<0.001**
Agitated	−1.85 (−2.88 to −0.82)	0.53	**<0.001**	−1.97 (−3.02 to −0.93)	0.53	**<0.001**
Feeding	2.0 (0.25–3.76)	0.90	**0.025**	1.93 (0.16–3.69)	0.90	**0.030**
Level 3						
Readers						
Research Nurse	Baseline			Baseline		
Observer 1	0.53 (0.19–0.86)	0.17	**0.002**	0.52 (0.19–0.85)	0.17	**0.002**
Observer 2	0.78 (0.44–1.11)	0.17	**<0.001**	0.78 (0.44–1.12)	0.17	**<0.001**

The multivariate analysis was performed using the variables with *P* values <0.05 from the univariate analysis. Final model included age (as continuous variable), temperature at baseline, history of rapid breathing, the child's state at the time of measuring the respiratory rate and readers. Intraclass variation = 87%. Bold *P*‐values indicates significant associations

The multivariate analysis showed no confounding between significant variables (Table 4). The multivariate linear regression model, with the three levels of variation (child, time and observer) explained 87% of the observed variation in the respiratory rates, leaving 13% of the variability as random error between the observers (research nurse, paediatrician 1 and paediatrician 2). Fifty‐three percentage of the variability between RR readings was due to the differences between children, and 34% due to different RR over time within the observed children.

## Discussion

We aimed to assess the use of chest movement video recordings of children presenting to a busy outpatient department in order to assess the use of videos as a reference standard of paediatric respiratory rate. We used two main indicators of success; did video provide a readable result and, if so, could experienced paediatricians agree on the ensuing respiratory rate? The results demonstrated that almost one‐fifth of videos did not provide a sufficiently clear view to count respirations, but when it did, agreement between expert readers was perfect (kappa = 0.85), indicating there was minimal misclassification of the WHO criteria for ‘raised respiratory rate for age’. However, there was only fair agreement on the actual respiratory rate measurements within 2 breaths per minute (kappa = 0.34), perhaps indicating the limitations of using a fixed tripod for video recordings of respiratory rate measurements of children in this setting.

There is clearly a need for training and quality control tools in paediatric respiratory rate given evidence of between‐observer variation in counting of respiratory rate in children with respiratory symptoms that can result in misclassification of children into the category of ‘fast‐breathing pneumonia’ 6, 14, 15. Two studies from Tanzania demonstrated only moderate levels of agreement between health workers that are likely to lead to both over‐ and undertreatment of childhood pneumonia 6, 14. However, these studies relied on direct observation and not video recordings. The findings have potentially important public health consequences as ‘fast‐breathing pneumonia’ is often the commonest reason for prescribing antimicrobial drugs in paediatric outpatients 16.

Video clips of paediatric clinical signs are an important source of teaching material although there are few validation studies of such videos. Among the problems of such validation is the fact that clinical signs often lack an objective measure that can act as a quality control tool and, as in our study, ‘agreement between experts’ has to act as the reference standard. A study to determine the level of interobserver agreement of video recordings of 11 common paediatric clinical signs found that lack of agreement among expert readers was often the result of the sign being ‘borderline’ or ambiguous 15. This is similar to the finding of our study, although respiratory rate, being counted on a continuous scale, may have less scope for interpretation but relies on a clear view of the chest movements of the child. Of relevance to clinical decision‐making, the agreement on the presence or absence of raised respiratory rate was particularly good.

We analysed clinical data to identify factors associated with readability of video recordings. Longer duration of illness (≥5 days) was the most significant child characteristic associated with readable video, and agitation of the child made a readable video less likely; the difference however was not statistically significant. The latter is consistent with an earlier study to determine variability in RR measurements over time which found that RR taken when the child is agitated is the most variable and is associated with largest number of failures 17.

The high ICC obtained in our study is in accordance with the results of another recent study conducted among Mozambiquan children with the aim of determining the reliability of RR assessment counted three times during a whole minute by independent observer paediatrician 18.

The two video observers in our study recorded higher RR than the direct measurements of the nurse, and interobserver differences accounted for 13% of the difference in RR measurements. The results show little confounding between variables, as the adjustment for the observer differences does not affect the coefficients for the other variables. Our results showed that half of the variability in the RR was due to differences between the children and a further third was due to differences over time within children.

### Study limitations

Videos were all taken from the same fixed position to provide the best view of where children lay on the couch. However, an improved view may have been possible by adjusting the camera position and future studies might explore this. The fact that the nurse operated a fixed camera and also did the counting of respirations may have confounded the RR counts of the nurse even though this was not a common occurrence as any disturbance led to repeat or discontinuation of the recording and RR count. Similarly, videos were taken at predefined time intervals as the primary aim of the study was to document how respiratory rate varies after placing children in a quiet environment designed to produce maximum settling effect. We recognise that variation in body temperature may influence respiratory rate and this could have influenced the readability of video records 4. Another limitation was that although we have used consultant paediatricians as a reference standard, they were not retrained in IMCI (WHO) respiratory rate count standards.

## Conclusion

Video recordings of respiratory rate in African children are constrained by the difficulty in obtaining a clear view of respiratory movements in up to one‐fifth of children with suspected pneumonia. More flexible use of the camera position may improve on this. Where a clear view is obtained, video is a valid tool for staff training and quality control purposes in clinical research. Future studies are needed to refine the methods used in our study.
